# Integrative medical group visits for patients with chronic pain: results of a pilot single-site hybrid implementation-effectiveness feasibility study

**DOI:** 10.3389/fpain.2023.1147588

**Published:** 2023-09-27

**Authors:** Isabel Roth, Malik Tiedt, Vanessa Miller, Jessica Barnhill, Aisha Chilcoat, Paula Gardiner, Keturah Faurot, Kris Karvelas, Kenneth Busby, Susan Gaylord, Jennifer Leeman

**Affiliations:** ^1^ Program on Integrative Medicine, Department of Physical Medicine and Rehabilitation, School of Medicine, University of North Carolina at Chapel Hill, Chapel Hill, NC, United States; ^2^ Gillings School of Global Public Health, Injury Prevention Research Center, University of North Carolina at Chapel Hill, Chapel Hill, NC, United States; ^3^ Department of Family Medicine, University of Massachusetts Medical School, Worcester, MA, United States; ^4^ Division of Pediatrics Hematology Oncology, Department of Pediatrics, School of Medicine, University of North Carolina at Chapel Hill, Chapel Hill, NC, United States; ^5^ School of Nursing, University of North Carolina at Chapel Hill, Chapel Hill, NC, United States

**Keywords:** chronic pain, integrative medicine, group medical visits, hybrid design, implementation mapping

## Abstract

**Background:**

Approximately 20% of adults in the United States experience chronic pain. Integrative Medical Group Visit (IMGV) offers an innovative approach to chronic pain management through training in mindfulness, nutrition, and other mind-body techniques combined with peer support. To date, there are no studies on IMGV implementation, despite its promise as a feasible non-pharmacological intervention for chronic pain management. In this study, we assessed the feasibility of implementing IMGV and assessing its effectiveness for chronic pain.

**Methods:**

Implementation Mapping was used to develop and evaluate implementation strategies for IMGV. Strategies included disseminating educational materials, conducting ongoing training, and conducting educational meetings. IMGV was delivered by three healthcare providers: an allopathic physician, registered yoga teacher, and naturopathic physician. The effectiveness of IMGV on patient health outcomes was assessed through qualitative interviews and a Patient-Reported Outcomes Scale (PROMIS-29). Provider perspectives of acceptability, appropriateness, and feasibility were assessed through periodic reflections (group interviews reflecting on the process of implementation) and field notes. Paired *t*-tests were used to assess changes between scores at baseline and post intervention. Qualitative data were coded by three experienced qualitative researchers using thematic content analysis.

**Results:**

Of the initial 16 patients enrolled in research, 12 completed at least two sessions of the IMGV. Other than fatigue, there was no statistically significant difference between the pre- and post-scores. Patients reported high satisfaction with IMGV, noting the development of new skills for self-care and the supportive community of peers. Themes from patient interviews and periodic reflections included the feasibility of virtual delivery, patient perspectives on acceptability, provider perspectives of feasibility and acceptability, ease of recruitment, complexity of referral and scheduling process, balancing medical check-in with group engagement, and nursing staff availability.

**Conclusions:**

IMGV was feasible, acceptable, and effective from the perspectives of patients and providers. Although statistically significant differences were not observed for most PROMIS measures, qualitative results suggested that participants experienced increased social support and increased pain coping skills. Providers found implementation strategies effective, except for engaging nurses, due to staff being overwhelmed from the pandemic. Lessons learned from this pilot study can inform future research on implementation of IMGV.

## Contributions to the Literature

• The Integrative Medical Group Visit (IMGV) program is a promising intervention for delivering evidence-based care for patients with chronic pain. Despite evidence of effectiveness and lower use of high-cost care among participants, IMGV has been minimally implemented.
• This is the first study to investigate implementation of IMGV. Due to the COVID-19 pandemic, IMGV was adapted to be delivered virtually via zoom. Here, we describe this adaptation, as well as implementation and effectiveness outcomes.
• These findings contribute to a gap in the literature on implementation of evidence-based integrative pain management interventions.

## Introduction

Over 50 million adults in the United States, or approximately 20%, experience chronic pain (1). To manage chronic pain, Clinical Guidelines encourage the use of non-pharmacologic therapies to mitigate potential adverse effects associated with opioid use and to improve overall patient care (2–4). The Department of Health and Human Services’ Pain Management Best Practices Inter-Agency Task Force Report specifically recommends complementary and integrative pain management services, including mindfulness-based stress reduction, yoga, acupuncture, movement therapy, art therapy, massage therapy, tai chi, biofeedback, and spiritual practice, as either stand-alone interventions or modalities included in a multidisciplinary approach to treat non-cancer-related acute and chronic pain (5).

Despite growing evidence on the effectiveness and importance of integrative pain management, limited accessibility, affordability, and pre-existing patient and provider assumptions complicate their implementation into clinical settings (6, 7). Barriers to accessing integrative pain management include patients’ and providers’ limited knowledge about these services and poor referral networks, among other factors (8, 9). Lack of affordability is compounded by the exclusion of integrative pain management services from most health insurance coverage (10).

The Integrative Medical Group Visit (IMGV) model is an innovative approach to integrative pain management, reimbursed by healthcare insurers, that offers training in mindfulness and other complementary approaches to chronic pain in a group setting with a healthcare provider. IMGV combines elements of existing group medical visit models, principles and practices of mindfulness-based stress reduction, and integrative approaches to chronic pain (11). IMGV was developed and tailored over the course of three years with the input of a Patient Advisory Board and Scientific Advisory Board to meet the needs of diverse, low-income patients with chronic pain (11, 12). The first single-arm IMGV study included eight weekly sessions and yielded significant reductions in pain (*p* = .005), depression (*p* < .001), sleep quality (*p* = .04), and perceived stress (*p* = .04) (*n* = 65). Qualitative findings included patients gaining a supportive network to share coping strategies for their chronic condition and feeling “not alone.” In a randomized controlled trial (RCT), a nine weekly session IMGV with booster (*n* = 76) was compared with a usual care control group (*n* = 79), and demonstrated significant between group differences (decreased pain medication use, decreased emergency department use, and improved mental health quality of life); however, recruitment and retention were noted challenges in conducting the trial (12).

These data provide evidence in support of the effectiveness of IMGV among participants who receive the intervention. Further study of the barriers and facilitators to IMGV implementation is needed to broaden IMGV reach and improve outcomes in populations at greatest risk for chronic pain. In this pilot study, we assessed the feasibility of implementing an IMGV and measuring its effects on chronic pain in an outpatient setting with a high burden of patients with chronic pain. By simultaneously studying effectiveness and implementation in a hybrid design, we can speed efforts to close the research-to-practice gap for pressing public health crises (13).

## Methods

### Setting

This pilot study took place in the UNC Health Center for Rehabilitation Care (CRC). The CRC is the primary outpatient clinical site for the UNC Department of Physical Medicine and Rehabilitation and the UNC Program on Integrative Medicine. In a recent survey of patients seen at the CRC, patients most frequently identified chronic pain as their primary complaint (14). Previous research conducted with patients at the CRC found that patients were interested in receiving integrative health services in conjunction with their established healthcare at UNC (15). Patients also wanted services that were affordable and accessible. Considering this feedback, IMGV was selected as a clinical model to pilot, as previous research on the model indicated that it was affordable, accessible, designed for diverse, low-income patients dealing with chronic pain, and offered integrative care.

### Intervention

IMGV, as developed by Gardiner et al. 2017, consists of nine weekly 2.5 h sessions, with a tenth follow-up session 3 months later (16). During the sessions, patients with chronic pain and depression participate in a group meeting and meet one-on-one with a clinician. Sessions are delivered by a billing medical provider (i.e., MD, DO, NP, PA) and a trained mindfulness or yoga instructor. Sessions begin with a go-around, where patients check-in about how they are doing based on a prompt. This is followed by discussion of a health education topic (i.e., ways to respond to stress, the importance of healthy sleep, our bodies and inflammation) and an experiential mind-body activity (i.e., mindful eating, chair yoga, self-acupressure, self-massage). Sessions conclude with a healthy meal shared by all, with recipes included in the participant manual. Participants are encouraged to incorporate these activities into their daily lives, and to practice mind-body techniques throughout the week.

In this pilot study, IMGV was delivered by a medical doctor trained in family medicine, a registered yoga teacher, and a naturopathic physician. Patients completed an initial one-on-one visit with the physician co-facilitator, who determined that they were eligible and invited them to participate in the IMGV. The physician also asked for their verbal consent to share contact information with the research team to confirm their initial interest in participating in the research study. Patients who were eligible to participate in the IMGV could participate in the group sessions without taking part in the research component. Due to the COVID-19 pandemic, all recruitment was conducted virtually and IMGV was delivered via telehealth using the HIPAA-compliant version of Zoom teleconferencing software. Zoom was chosen due to the ease of using breakout-rooms for one-on-one visits with the physician. For telehealth delivery, IMGV was shortened to 2 h (from 2.5 h). For this pilot, the 3-month follow up (tenth session) was removed.

The following adaptations were made prior to recruiting the first cohort:

1. The IMGV participant and facilitator manuals were reviewed and edited by the study team to ensure that language in the curriculum related to physically attending a live session was removed or adapted to fit the virtual setting.
2. Hard copies of IMGV participant manuals were mailed to participants and they had access to an online version as well as a website with supplemental materials.
3. Participants received individual email meetings and a zoom link containing the meeting information prior to the first IMGV session.
4. Participants were called the day before and an hour prior to the first session to address technical challenges and to ensure access to the IMGV manual.
5. The healthy meal at the end of each session was replaced with a cooking demonstration of the recipes included in the participant manual.

### Implementation strategies

The selection and tailoring of implementation strategies for IMGV was guided by implementation mapping (17). During this process, researchers engaged clinic stakeholders (clinicians, nurses, and administrators) to identify barriers and facilitators to implementation (18). The research team then selected relevant theories, including social cognitive theory and diffusions of innovation theory that were used together with a pre-existing implementation strategy taxonomy, and the Consolidated Framework for Implementation Science 2.0 (CFIR 2.0) to organize and execute implementation efforts (see Table 1) (12, 18–21).

**Table 1:** Implementation strategies.

Implementation strategy [Named using the ERIC taxonomy] (21)	Definition [Definitions from ERIC taxonomy] (21)	Actor who enacts the strategy	Action	Action target [Action targets are specified using definitions from CFIR 2.0] (24)	Temporality	Dose	Implementation outcome affected
Conduct ongoing training	Plan for and conduct training in the clinical innovation in an ongoing way	Innovation expert	Provide didactic and experiential activities (roleplay) to train innovation deliverers (IMGV co-facilitators)	Implementation process; innovation deliverers teaming and planning knowledge and self-efficacy of innovation deliverers to deliver innovation	Prior to beginning implementation activities	3 day in-person training	Effectiveness, fidelity
Conduct educational meetings	Hold meetings targeted toward different stakeholder groups to teach them about the clinical innovation	Innovation deliverers	Meet with/present to referring depts./clinicians; Including grand rounds, one-on-one meetings, and invitations to shadow IMGV sessions	Leadership and staff capability, opportunity, and motivation to refer patients to IMGV and support implementation	Before groups begin, then bimonthly	6 meetings and presentations	Reach
Distribute educational materials	Distribute educational materials (including guidelines, manuals, and toolkits) in person, by mail, and/or electronically	Innovation expert	Mail IMGV facilitator and participant manuals to clinic	Implementation process; available resources; materials and equipment and access to knowledge and information to deliver IMGV	Before groups begin	Once	Effectiveness, fidelity
Provide local technical assistance	Develop and use a system to deliver technical assistance focused on implementation issues using local personnel	Innovation deliverers	Identify and prepare Information technology experts to troubleshoot implementation issues for IMGV; reserve telehealth platform and create private meeting room	Inner setting; structural characteristics information technology infrastructure	During implementation activities, before groups begin	As often as needed throughout implementation activities (may range from multiple times per week to none)	Feasibility

Table 1 Implementation strategies were selected based on determinants identified through stakeholder interviews. Implementation strategies are defined using the Expert Recommendations for Implementing Change (ERIC) taxonomy and specified using Proctor’s recommendation for specifying implementation strategies (12, 22).

### Outcome measures

Outcome measures were chosen based on both the RE-AIM framework and Proctor’s Outcome Measures (23, 24). To assess the impact of the implementation strategies, we assessed reach, effectiveness, and implementation.

#### Reach

 Reach was assessed as the percentage and demographics of those referred to IMGV who enrolled versus declined. Demographic information included age, race, ethnicity, gender identity, and insurance status. Engagement was measured by attendance at IMGV sessions.

#### Effectiveness

To assess effectiveness of the intervention, patient perspectives were assessed through open-ended questions, and health outcomes through a Patient-Reported Outcomes measure, the PROMIS-29, which was administered by a research assistant (RA) over zoom or telephone before and after participation in the telehealth IMGV program. Participants were given a $20 gift card as an incentive for completing each interview. The PROMIS-29, a comprehensive measure of pain and related quality of life measures, was administered before and after completing the 9-week program. The PROMIS-29 has been shown to have high reliability and validity in the general population (25).

#### Implementation

Provider perspectives of acceptability, appropriateness, and feasibility were assessed through periodic reflections (group interviews reflecting on the process of implementation) and field notes (26). The RA led three periodic reflections, conducted via zoom. Participants included the IMGV physician, yoga teacher, naturopathic physician, a referring physician, and co-investigators. Periodic reflections were conducted using a semi-structured interview guide focused on barriers and facilitators to implementation as well as feasibility of the selected implementation strategies.

### Analysis

Descriptive statistics were performed on quantitative data. Univariate analysis was performed to check for normality among continuous variables. Means and standard deviations were calculated by treatment phase (baseline and post intervention) and frequency data (n and percent) is reported for categorical variables and demographic data. Paired *t*-tests were used to assess statistically significant changes between scores at baseline and post intervention to control for within-individual variability. In addition to paired *t*-tests, Wilcoxon Signed-Ranks Tests were also selected to assess changes in pain ratings and PROMIS measures measured at two time points (Supplementary Table A). All analyses were performed using SAS 9.4 (29).

Qualitative data, including patient interview transcripts and periodic reflection transcripts, were coded by three experienced qualitative researchers (IR, SG, AC) using thematic content analysis (30). Dedoose qualitative data management software was used to manage and organize codes (31).

## Results

### Reach

**Figure 1:**
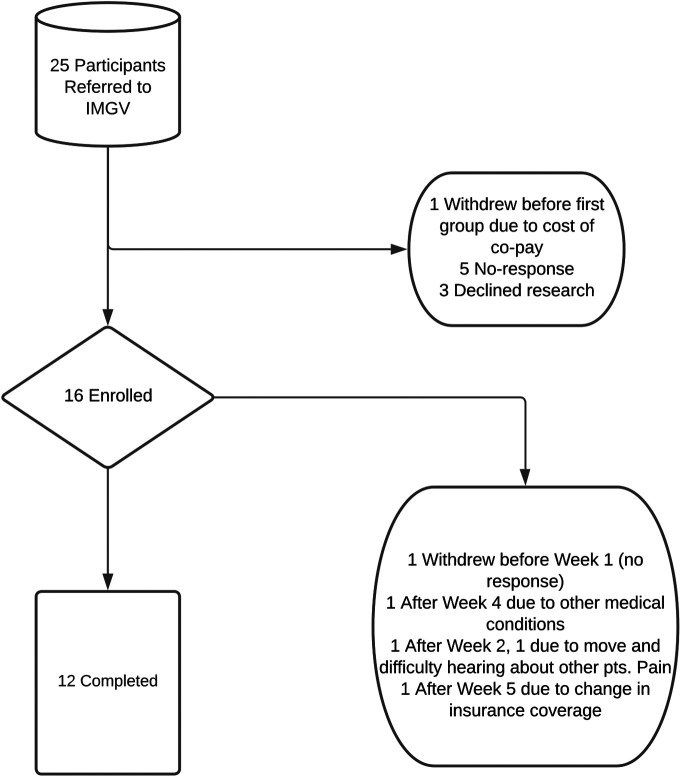
Describes the enrollment process of referral for integrative medical group visits. Nine participants chose not to enroll, while 4 chose to withdraw from the study itself. 16 participants enrolled, and a total of 12 participants completed the study.

A total of 25 patients with chronic pain were referred to participate in the IMGV program (see Figure 1). Sixteen patients consented to participate in the research study. Fourteen participants completed pre-interviews; twelve attended more than two IMGV sessions and completed post-interviews.

Of the four participants who dropped out of the IMGV, two (50%) were Black or African American, one (25%) was Asian or Pacific Islander, and one (25%) was White. Regarding insurance, three (75%) participants were enrolled in Medicaid, two (50%) in private insurance, one (25%) in Medicare, and one (25%) held Military (Veteran’s Administration) coverage. The average age of participants who dropped out was 55, ranging from 37 to 76 years old. One patient was lost to follow up after the initial Zoom orientation and never attended an IMGV session, one participant withdrew after week two because they were moving homes and had difficulty hearing about other patients’ pain, one patient attended two of the first four sessions and then withdrew due to unrelated health issues, and one patient withdrew after week five due to a change in their insurance coverage (and related inability to afford the new co-pay).   

Table 2 describes the age, gender, insurance status, and race demographics of patients who completed both pre and post interviews and completed the IMGV.

**Table 2:** Demographics of participants completing research activities (*N *=* *12).

	Count (*n*)	Frequency (%)
Age		
Mean (SD)	51.6 (12.3)	
Range	31–70	
Gender		
Female	10	83.3
Male	2	16.6
Insurance		
Medicaid	3	25.0
Medicare	2	16.6
Private	5	41.6
Military/VA	0	0.0
Charity care	1	8.3
None	2	16.6
Race		
Black or African American	2	16.6
White or European American	9	75.0
Asian or Pacific Islander	1	8.3

Of these patients, 83.3% were female, 75% were white, 16.6% were Black or African American, and 8.3% were Asian or Pacific Islander. No patients identified as Spanish, Hispanic, or Latino/a (0%). The mean age was 52.6, ranging from 31 to 70 years. A total of 41.6% were enrolled in private insurance, 25% in Medicaid, 16.6% in Medicare, 16.6% were uninsured, and 8.3% were covered by the health system’s financial aid program (multiple patients were dually-eligible).

**Figure 2:**
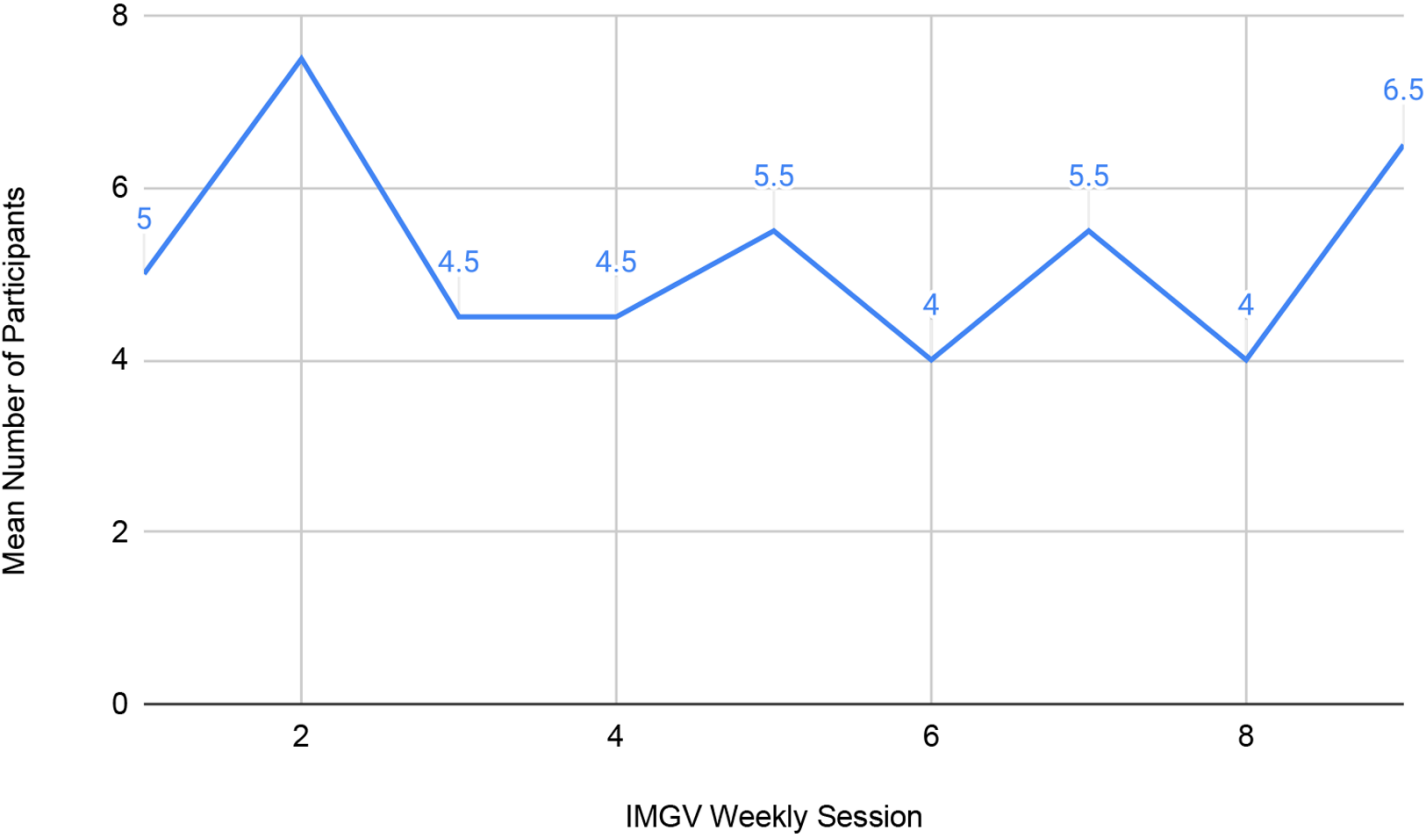
Describes average participant attendance in the IMGV over the course of 9 weeks.

Over the course of two nine-week cohorts, each session was attended by an average of five patients per session (see Figure 2). Nine patients were enrolled in cohort 1 and seven in cohort 2.

Attendance at each session ranged from two to nine attendees, with higher overall attendance in cohort 1 (6.44) than cohort 2 (4.00). Cohort 1 ran continuously from late August through early November of 2020. Cohort 2 ran from November of 2020 through February of 2021, with breaks for Thanksgiving and winter holidays.   

### Effectiveness

Results of the paired *t*-tests found a statistically significant difference in one measure: an increase of 3.98 points (95% CI 0.52, 7.43) on the fatigue subscale of the PROMIS-29 (*M* = 56.69, SD = 12.44 and *M* = 60.67, SD = 10.46 for pre-IMGV and post IMGV, respectively), *t*(11) = 2.53, *p* = 0.03. Physical function scores increased as did depression, fatigue, and satisfaction with social roles. Anxiety, sleep disturbance and pain interference decreased. However, other than fatigue, there was no statistically significant difference between the pre- and post-scores. This finding was supported by the non-parametric Wilcoxon Signed-Rank test results (Supplementary Table A).

Four individuals (33%) experienced a decrease in their pain score when the post-IMGV rating was compared with the pre-IMGV rating. The largest difference was a decrease of 3 (a pre-IMGV pain rating of 10 and post-IMGV rating of 7). This individual also experienced an increase in physical functioning, anxiety and pain interference while reporting an increase in depression, fatigue, sleep disturbance and satisfaction with participation in social roles. The three other people who experienced decreases in pain rating all exhibited a decrease of one point on the pain scale. Three people experienced no change in their pain rating, all three people rated their pain as a 7 at both pre and post measurements. Five people reported a higher pain rating after IMGV, with the largest increase being a 2-point increase (from 6 to 8). This individual had no change in physical functioning, pain interference, fatigue or sleep disturbance, increased anxiety, depression, and a decrease in satisfaction with participation.

Table 3 describes PROMIS-29 subscale scores before and after participation in an IMGV.

**Table 3:** Pre-and post- mean scores for pain rating (1–10) and PROMIS measures with 95% confidence intervals from paired *t*-tests, *n *=* *12.

	Before intervention mean (95% CI)	After intervention mean (95% CI)	Mean difference (95% CI)
Pain rating (1–10)	6.5 (5.4, 7.6)	6.5 (5.6, 7.4)	0.0 (−0.9, 0.9)
Physical functioning	38.9 (36.2, 41.7)	40.2 (37.4, 42.9)	1.3 (−0.7, 3.2)
Anxiety	64.5 (59.7, 69.3)	64.1 (59.8, 68.3)	−0.5 (−4.8, 3.9)
Depression	58.8 (53.1, 64.5)	61.0 (59.9, 65.1)	2.2 (−2.2, 6.5)
Fatigue	56.7 (49.3, 64.1)	60.7 (54.4, 66.9)	4.0 (0.5, 7.4)
Sleep disturbance	56.1 (51.4, 60.8)	55.3 (51.4, 60.8)	−0.8 (−4.5, 2.9)
Satisfaction with participation in social roles	50.3 (45.4, 55.2)	51.3 (48.0, 54.6)	1.0 (−4.4, 6.4)
Pain interference	69.0 (65.8, 72.3)	68.3 (65.7, 70.9)	−0.7 (−2.9, 1.4)

For PROMIS measures: a score of 50 is the standard population level mean, and an increase/decrease of 10 represents 1 standard deviation. In this sample, the mean physical functioning score is almost 1 standard deviation lower than the population mean, indicating physical functioning is low in this group. The summary scores as extreme as 1 standard deviation from the normal are the pain interference and anxiety scores, indicating this sample has higher pain interference and higher anxiety than the population level mean. All scores reference the last 7 days except the physical functioning score which has no temporal anchor.

#### Patient perspectives on how and why IMGV was effective

Qualitative themes from patient interviews included that the IMGV provides connection with others who can understand chronic pain, participants gain new tools to cope with chronic pain, and participants value the positive group environment. Patients expressed that the information and skills gleaned from the group were helpful, even if they didn’t eliminate pain. One patient said, “the meditation and the yoga itself was a little bit of a physical release. It doesn’t exactly make the pain go away but it makes it easier to deal with.” She went on to describe the components of the program that were helpful.

“One was just the camaraderie. I had people to talk with and get to know who’s had similar problems. I got a lot of good information on nutritional eating. I’ve gotten a lot of help with learning to do meditations and that has been very helpful to me. The yoga that we know has been very helpful to me.”-pt.1015

Some patients noted that specific tools helped their functional abilities.

“…Other ways to help ease my pain. The nutrition information, foods that were good. The proteins, what’s good for inflammation, the chair yoga. I've been doing more. At first it hurt, but I've tried each day and it is getting better and easier to turn. The acupressure really helps my neck.”-pt. 1011

For many patients, being able to have improved quality of life without adding new medications felt important, particularly in light of the challenges associated with many pain medications.

“I got some new tools and ways that I can- things that I can do to help me feel better without having to do any medications.”-pt. 1004

Patients particularly appreciated the social support from others who could understand their experiences.

“I think that part of it was good because there was communication between other chronic pain patients, and I think that that’s really good for chronic pain people…it’s nice to connect with people who have the same thing”-pt. 1010

### Implementation

#### Patient perspectives on feasibility of virtual delivery

Virtual delivery of the IMGV proved to be incredibly helpful for some patients, who noted that they were able to participate on days when they were in so much pain that they wouldn’t have otherwise been able to leave the house.

“The big plus for me being virtual was that I'm currently not really able to drive. I don't have–If it is one of those situations where I would have to rely on Uber or Lyft or my husband to get me to a weekly session, that probably would have not been possible. One of the benefits was I could sit on my bed and log into the computer and participate”-pt.1012

Participants would regularly attend the IMGV and note that they were having a particularly hard day. Some patients did not have their cameras on for the full session when they felt this way. Others noted that on days when they were experiencing a lot of pain, it was difficult to focus on the session content.

“Just trying to sit and the chronic pain does interrupt your thought processes. At certain points, my brain would be focused on the pain instead of focused on the class. There was a little bit of that during the second hour each week.”-pt. 1010

This presents a challenge for any behavioral intervention for people in pain—it is difficult to focus or be active during pain flare-ups. Others noted that it would have been more comfortable if sessions had been held in-person.

“It was difficult doing it through the computer…I prefer actual in a group versus online. It was a little uncomfortable, you don't see everybody, and you don't really see what’s going on in the space. I guess, I'm used to seeing something different like what I saw in [a group program for substance use disorder]. It was just a little uncomfortable, but not uncomfortable enough to make me not want to participate.”-pt. 1009

#### Patient perspectives on acceptability

Overall, patient participants found the IMGV highly acceptable, and advocated that the University health system continue the program. One patient said,

“My general takeaway is that it was a wonderful program…I would like to be able to see UNC continue groups like this, even if it’s not part of a study, because it was a very good experience, and the support system was just very beneficial. I know I was sad when it was over. I was asking, are you going to start a second group? I want to be part of a second group!”-pt.1012

At the same time, she went on to note that the curriculum material was not equally applicable across all chronic pain conditions. She said,

“I think some of the material was helpful, but sometimes it was hard for me to participate because the program seems to be aimed at people who have chronic pain from inflammation, like chronic arthritis or something and for degenerative disc disease or something. The type of chronic pain that I have really doesn't fit in that category. So some of the sessions and some of the material presented really didn't fit my particular needs.”

Other patients noted that the IMGV was well-structured and wouldn’t have wanted to change it.

“I thought the program was well structured with just enough accountability without being pushy or overly aggressive on doing the homework. It was perfect, I thought the way it was structured was perfect.”-pt. 1004

#### Provider perspectives of feasibility and acceptability

Providers noted the ease of recruiting patients into the program, the complexity of setting up a new referral and scheduling process, the challenge of balancing the medical check-in portion of the IMGV with group engagement, and that nursing staff had conflicting clinical roles that prevented participation in the program [5 months into the COVID-19 pandemic].

##### Ease of recruitment

Despite launching recruitment efforts during the first year of the COVID-19 pandemic, recruitment into the IMGV proceeded smoothly. The IMGV physician was familiar with the patient population at the clinic and recognized a need for interventions for patients with chronic pain. As implementation efforts got underway, she perceived that the main need was to inform other clinicians that the program was happening.

“I’m not incredibly worried about recruitment, but I do think it’ll be important, at least within our department, to tell everybody this is happening so that there’s at least the option of recruiting those patients. Starting a wait list, I think, would actually be helpful for assisting in the program.”-IMGV Physician

A flyer was developed and approved by the department leadership and emailed to department staff and faculty. Other than developing the flyer and emailing it out to the clinic staff, there were relatively few other staff engagement activities planned, as the clinic was understaffed and staff were short on time, and many others were working from home at the time.

“Yeah, so we did complete—the flyers were approved and completed. I think that making sure that those flyers are up and that [clinic] staff have access to them…I sent in that email to the [department] listserv talking about the group, but that’s really the only effort that I’ve made to involve staff.”-IMGV Yoga Teacher

Another recruitment effort included giving a departmental grand rounds on the program, though due to scheduling issues this was done late in the process. Additionally, referring physicians were invited to participate in a group session to have direct experience and improve their initial communication with their patients for recruitment. One referring MD did do this and enjoyed the interactive role during that session, especially seeing known patients being engaged.

Faculty physicians began referring patients with chronic pain to the IMGV physician, who then scheduled individual visits with patients to discuss integrative medicine approached to chronic pain and assess interest in group-based care. During this phase, few barriers were encountered when recruiting patients in the IMGV. Patients were interested in the model and were looking for new approaches to manage their chronic pain conditions. The IMGV physician was excited to begin the groups and that patients expressed interest in participating.

##### Complexity of referral and scheduling process

One of the challenges the implementation team faced was developing a simple referral process for the IMGV in the electronic health record. The team members noted that referrals for patients with pain could sometimes be confusing, noting that referrals did not always specify which physician the patient was being referred to see. In addition to IMGV, the physician offered integrative medicine consults at the clinic. This began during the same time period and there was confusion among staff about who should be referred to integrative medicine and what to tell patients to expect. The implementation team realized that they needed the collaboration of the scheduling team and the embedded electronic health record specialist to develop a referral process to easily direct patients to the IMGV.

“there’s some step there of getting people on to my schedule. I don’t think I have access rights in [the electronic health record] to do that. We’ll need someone to do that.”- IMGV Physician

In addition to developing a process to easily direct referrals to the IMGV physician, a new template needed to be built so that multiple patients could be scheduled to see the same physician at the same time.

“There was building a new scheduling template so that [IMGV Physician] could see 10 patients at a time, and a lot of back and forth with the scheduling staff about how to communicate with them.” -IMGV Yoga Teacher

While developing this electronic infrastructure required some initial set-up and explanation of the group visit model to the clinic staff, once the referral process and scheduling templates were in place, there were few hiccups.

##### Balancing medical check-in with group engagement

To deliver the groups online, the implementation team needed to re-design the medical check-in portion of IMGV. When sessions had been held in-person in previous studies, patients were handed a check-in sheet which they filled out and handed to the doctor. The doctor was then able to review patient’s check-in sheets with them quickly and pull them aside one-on-one, or patients could share their check-in with the group. For virtual delivery, patients were given a brief survey to fill out weekly for the medical check-in. This facilitated the physician being able to address any pressing medical questions with the participants. However, it also became apparent that participants felt comfortable completing their check-ins with the group, and only wanted to meet with the physician one-on-one to discuss particularly private or complex topics. Many patients wanted to share updates related to their chronic pain with other members of the group weekly. The physician said,

  “Yeah, something for us to figure out, how to do this efficiently…the other thing we had asked was about, do we need to pull people out? The answer was no. The check-ins can happen in a Zoom group the way they can happen in-person in a group…with the group there’s a lot of sharing that happens”—IMGV Physician

The IMGV physician also noted that because the group was co-facilitated by a yoga instructor, she was able to complete notes while the group was going on. One-on-one check-ins with patients happened before and after the two hours of group time (and occasionally during the group time for pressing concerns), but overall, the physician felt that this was a manageable workload.

##### Nursing staff availability

One of the planned implementation strategies identified through the implementation mapping process was to train nursing staff to administer the check-in process for patients as they arrived for the IMGV. However, as the pandemic continued and it became clear that the only way to deliver a group intervention would be through telehealth, this strategy became less tenable. Shortly before the IMGV program was scheduled to begin, the nurse manager passed away unexpectedly, and the small team was left grief-stricken and shorthanded. Rather than ask the nursing team to take on the task of learning how to administer a new clinical intervention, the implementation team chose to take on the enrollment and check-in process themselves.

“I think we’re assuming that staff is pretty much overworked with the shortage and nursing and scheduling folks. We’re proceeding on the assumption that, basically, [yoga instructor] and I are gonna do all of that…I don’t think we’ll be asking for much from staff.”-IMGV Physician

While the implementation team had initially intended to manage the check-in process temporarily and involve the nursing staff more integrally once a new nurse was hired, it became clear over time that there were continuing demands on the small nursing team. Ultimately, while one nurse shadowed several IMGV sessions, nursing staff did not take on any role in the IMGV, and the facilitators managed the check-in process independently.

## Discussion

In this pilot study, IMGV was successfully implemented into an outpatient rehabilitation center. Ten of the eleven implementation strategies developed using the five tasks of implementation mapping were feasible and successfully executed. Patients and providers found the IMGV program highly acceptable and feasible. Quantitative measures of IMGV showed that physical functioning and satisfaction with participation in social roles increased, while anxiety, pain interference, and sleep disturbance decreased. However, fatigue significantly increased, and though not significant, depression increased as well. Qualitative findings indicated that patients found that IMGV gave them new tools to cope with their pain and social support from others who understood their unique circumstances. Interpretation of the PROMIS-29 changes may be linked to the onset of the COVID-19 pandemic and the disruption in activities due to the stay-at-home order in North Carolina.

The use of a hybrid implementation-effectiveness study design helped to create a holistic if complex picture of the process of implementation. To assess both implementation and effectiveness outcomes, this small pilot study involved several data capture methods, including patient interviews, periodic reflections, field notes, patient reported outcome measures, and recruitment, retention, and attendance data. While this was feasible, future larger trials with larger volumes of qualitative data may benefit from the use of rapid qualitative data analysis methods (30).

The IMGV program was initially designed for primary care settings, and this implementation context was distinct in that it was an outpatient rehabilitation clinic. Patients in this setting may present with more complex pain than average primary care patients, as indicated by the low physical functioning scores in this sample. The patients in this study represented a heterogeneous population; some people had experienced chronic pain for decades, some for less than 1 year, with a wide range of diagnoses.

There was substantial interest in IMGV from providers and patients, and a clear perceived need for non-pharmacologic, integrative resources for chronic pain management in this setting. However, only 12 of the 16 patients who initially enrolled in research on IMGV went on to complete the intervention. Previous studies of IMGV have similarly found that more patients initially express interest in the program than actually enroll and attend (12). In addition to reasons for dropout documented in other studies, such as challenges with scheduling, other medical issues, and family obligations, we found that some patients expressed difficulty listening to other patients talk about their pain (12). While the literature on group medical visits has noted that some patients prefer not to meet in groups, difficulty listening to others talk about pain may be a specific challenge for chronic pain IMGVs. Retention of participants was relatively high, with an average of 6 patients attending each session. This allowed for substantial enough groups to form cohesive communities and represent different demographics.

Overall, implementation of the program was successful, as the program was implemented, was well-received by participants and providers, and remains ongoing. Group visits remain a novel concept for schedulers and administrative systems, and there is a need to set up new systems and educate staff involved in how to adapt to new processes. Virtual delivery provided new opportunities for patient access, particularly for improving attendance for patients struggling with pain who expressed barriers to leaving their homes. Virtual delivery also created new challenges for those with low tech-literacy, the need for additional resources to maintain participant engagement, and some need to adapt the intervention and curriculum. It is difficult to determine from this small single-site observational pilot study if the chosen implementation strategies were particularly helpful, or if other implementation strategies might have been more effective.

### Limitations

The conclusions of this study are limited by its single site nature and small sample size. Further, data was collected from spring 2020-spring 2021, at a time when the COVID-19 pandemic was rapidly changing many facets of life and specifically healthcare in the United States and around the world. At the time, the clinical team was already feeling the nursing shortage that continues today (26, 30). This seemed to be a context-specific issue because the nurse manager at this clinic passed away at the start of the pandemic. However, low staffing ratios may have been part of larger national trends and nursing shortages. Nursing staff at this site were very busy before the pandemic, and the perceived stressors for this group only intensified as the pandemic unfolded.

The COVID-19 pandemic and subsequent lockdown also significantly altered many engagement activities. Working from home limited interactions with staff at the CRC, and IMGV did not take place in-person as initially planned. Adaptations to IMGV delivery were necessary due to constraints of the pandemic.

While the course curriculum was closely followed, this intervention differed significantly from the originally developed and tested program (11). This intervention tested one component, the group visit, as delivered in a virtual setting. The other components, specifically the embedded conversational agent, the meal served after the in-person group visit, and checking blood pressure and weight at the start of each visit, were not included. The IMGV Leaders followed the patient empowerment, non-hierarchical approach set forth in the IMGV manual and reinforced during training sessions at the Integrated Center for Group Medical Visits training in Lawrence, MA (29, 31). However, adaptations were substantial enough to limit utility of the fidelity checklist created by the intervention developers (11). Future studies of IMGV would benefit from the use of a fidelity tool assessing delivery of core components of IMGV.

Finally, a key limitation of this study is that sustainment strategies were not developed during the first tasks of implementation mapping. Implementation strategies focused on the pre-implementation and implementation phases and did not account for what would happen if IMGV implementation were successful. During the data collection phase, students and trainees contributed to the IMGV program delivery and data collection, however, a need remained for permanent staff to continue to engage participants (including calling and scheduling new patients). From the administrative lens, a budget impact analysis and/or cost effectiveness analysis would provide key information to decision-makers, as staff time and financing was of principal concern for sustaining the program.

### Innovation

To date, there have been no studies on implementation of IMGV specifically, despite its promise as a feasible non-pharmacological intervention for chronic pain management. Use of a hybrid implementation-effectiveness design allowed us to look at issues impacting implementation and effectiveness simultaneously. Using periodic reflections allowed for data capture while minimizing burden on busy clinicians and implementation staff. Incorporating multiple learners (student RA, post-doctoral fellows), allowed for a complex pilot study and data collection with a small budget. Finally, methods prioritized patient perspectives and preferences.

## Conclusion

There is substantial interest and need among patients with chronic pain and the healthcare providers who treat them to make integrative pain management programs affordable and accessible. Prior studies have established that IMGV is a promising model to improve physical functioning, decrease anxiety, and improve social satisfaction among patients with chronic pain in a format that is reimbursed by insurance companies. In this study, Implementation Mapping allowed for the development of theory-based implementation strategies evaluated using implementation outcomes. In this small, single-site pilot study, IMGV was successfully implemented into an outpatient rehabilitation center, with a population of patients with chronic pain with low physical functioning.

However, in this small sample, fatigue was the only sub-score of the PROMIS-29 to change significantly, and fatigue was significantly worse post-intervention. It is unclear from this pilot study if the intervention was not effective, the adaptations from the original intervention were too substantial, the measures being used were not responsive enough in the short time frame, or were not measuring the correct constructs. With a very small sample size, no control condition, and no randomization, limited conclusions can be drawn from this pilot study.

Lessons learned in this pilot study provide valuable insight for future, fully powered trials. Methods used in this pilot study could be replicated in future research with larger sample sizes, control conditions, and randomization to determine effective implementation strategies for IMGV. Future research should include multiple clinical sites with diverse patient populations, development of sustainment strategies, a cost evaluation component, measurement of fidelity to core components of IMGV, and use rapid qualitative analysis methods to act upon qualitative findings. While disruptive events like the COVID-19 pandemic cannot be predicted, future research should plan for some flexibility in implementation efforts, as well as thorough documentation of adaptations, given the unpredictability of world events.

## Data Availability

Data will be made available upon request subject to a data use agreement.
