# Supplementary material for: Integrative medical group visits for patients with chronic pain: results of a pilot single-site hybrid implementation-effectiveness feasibility study
Source: Front Pain Res (Lausanne). 2023 Sep 27;4:1147588. doi: 10.3389/fpain.2023.1147588 (PMC10565345; doi:10.3389/fpain.2023.1147588)
Supplement: Supplementary file 1 [file Table1.docx]

Supplementary Table A. Wilcoxon z-scores and p-values for non-parametric Wilcoxon rank-sum tests of paired pre- and post-scores.

| Variable | Wilcoxon z-score | P-value |
| --- | --- | --- |
| Pain rating (1-10) | 0.2 | 0.918 |
| Physical functioning | 1.1 | 0.288 |
| Anxiety | -0.3 | 0.777 |
| Depression | 1 | 0.359 |
| Fatigue | 2.6 | 0.008 |
| Sleep disturbance | -0.7 | 0.531 |
| Satisfaction with participation in social roles | 0.2 | 0.879 |
| Pain interference | -0.7 | 0.527 |
